# Eukaryotic diversity at pH extremes

**DOI:** 10.3389/fmicb.2012.00441

**Published:** 2013-01-17

**Authors:** Linda A. Amaral-Zettler

**Affiliations:** ^1^Marine Biological Laboratory, Josephine Bay Paul Center for Comparative Molecular Biology and EvolutionWoods Hole, MA, USA; ^2^Department of Geological Sciences, Brown UniversityProvidence, RI, USA

**Keywords:** acidophiles, alkaliphiles, pyrosequencing, protists, V9, indicator OTU analysis

## Abstract

Extremely acidic (pH < 3) and extremely alkaline (pH > 9) environments support a diversity of single-cell and to a lesser extent, multicellular eukaryotic life. This study compared alpha and beta diversity in eukaryotic communities from seven diverse aquatic environments with pH values ranging from 2 to 11 using massively-parallel pyrotag sequencing targeting the V9 hypervariable region of the 18S ribosomal RNA (rRNA) gene. A total of 946 operational taxonomic units (OTUs) were recovered at a 6% cut-off level (94% similarity) across the sampled environments. Hierarchical clustering of the samples segregated the communities into acidic and alkaline groups. Similarity percentage (SIMPER) analysis followed by indicator OTU analysis (IOA) and non-metric multidimensional scaling (NMDS) were used to determine which characteristic groups of eukaryotic taxa typify acidic or alkaline extremes and the extent to which pH explains eukaryotic community structure in these environments. Spain's Rio Tinto yielded the fewest observed OTUs while Nebraska Sandhills alkaline lakes yielded the most. Distinct OTUs, including metazoan OTUs, numerically dominated pH extreme sites. Indicator OTUs included the diatom *Pinnularia* and unidentified opisthokonts (Fungi and Filasterea) in the extremely acidic environments, and the ciliate *Frontonia* across the extremely alkaline sites. Inferred from NMDS, pH explained only a modest fraction of the variation across the datasets, indicating that other factors influence the underlying community structure in these environments. The findings from this study suggest that the ability for eukaryotes to adapt to pH extremes over a broad range of values may be rare, but further study of taxa that can broadly adapt across diverse acidic and alkaline environments, respectively present good models for understanding adaptation and should be targeted for future investigations.

## Introduction

Life thrives at many extremes, but how eukaryotes adapt and diversify in these environments remains underexplored. Extreme pH environments are typically described as those possessing a pH less than 3 and greater than 9 (Gross and Robbins, 2000; Horikoshi, 2004). Both acidic and alkaline pH extreme environments often include other abiotic extremes as well (Rothschild and Mancinelli, 2001). Eukaryotes that thrive at acidic extremes are often subjected to not only orders of magnitude differential concentrations of hydrogen ions outside their cells, but also high concentrations of toxic metals (Whitton, 1970), low nutrient levels (Brake and Hasiotis, 2010), and/or extreme temperatures (Brock, 1978). Likewise extreme temperatures and salinities often characterize extreme alkaline environments (Horikoshi, 2004).

Both extremely acidic and extremely alkaline environments can be attributed to natural and anthropogenic sources. Classic examples of acidic environments include acid rock drainage (ARD) or acid mine drainage (AMD) systems often involving current or past mining activities, hydrothermal vent fluids, and terrestrial geothermal environments. Alkaline environments include soda lakes, hot springs, hydrothermal vent systems, as well as environments shaped by industries such as papermaking and textiles. In general, acidophilic eukaryotic diversity is more extensively studied, probably due to a larger number of accessible environments characterized by these extremes and relevance to biotechnological applications. Examples include ARD (Amaral Zettler et al., 2002) and AMD environments (Baker et al., 2003), acidic geothermal springs (Aguilera et al., 2010) and acidic lakes (Brown and Wolfe, 2006). Several well-known pH extreme environments such as alkaline Mono Lake in California, have been extensively examined for their bacterial and archaeal diversity but remain underexplored with respect to microbial eukaryotic diversity (Hollibaugh et al., 2001; Humayoun et al., 2003). Others such as acidic Nymph Creek in Wyoming have been the focus of targeted (Sheehan et al., 2003; Ferris et al., 2005) microbial eukaryotic work but have never been surveyed for overall eukaryotic diversity.

Despite the wealth of studies that focus on individual extreme environments, comparisons across multiple extremes are rare. Studies of eukaryotes in extremely acidic environments have revealed representatives from multiple evolutionary lineages, suggesting that the ability to adapt to pH extremes may be widespread (Amaral Zettler et al., 2002; Costas et al., 2007; Lopez-Rodas et al., 2008, 2011). This raises the question of whether there are cosmopolitan eukaryotic taxa that have adapted to a wide range of pH extremes. Which environmental parameters are most influential in shaping eukaryotic microbial diversity patterns at pH extremes also remains underexplored.

Investigations of eukaryotes at pH extremes have a long history (Brock, 1978), but an obstacle to unveiling the extent of eukaryotic diversity at pH extremes is that many of these environments have low evenness with a small number of species that make up biofilm communities dominating a given site. A lack of consistent taxonomic coverage in biodiversity inventories has also hindered comprehensive morphology-based surveys in general. It was not until the advent of molecular approaches employing general eukaryotic primers that the extent of eukaryotic diversity at pH extremes was more fully revealed. The application of next-generation DNA sequencing has helped to unveil the rarer forms of eukaryotes that exist behind the backdrop of more abundant forms (Stoeck et al., 2010). Yet, despite efforts to catch up with the bacterial and archaeal efforts, investigations of eukaryotic diversity at pH extremes and extreme environments in general still lag behind those of other domains. A major challenge to quantifying eukaryotic diversity is PCR-bias associated with the sometimes extreme length variation in ribosomal RNA (rRNA) genes. Another challenge (seldom acknowledged) is accurately representing the relative abundance of a given eukaryotic species or phylotype in the environment, because microbial eukaryotes can have copy numbers of their rRNA genes that differ by 4 orders of magnitude. This confounds our ability to apply abundance-based metrics in quantifying eukaryotic diversity in natural environments. Techniques such as fluorescence *in situ* hybridization (FISH) offer a solution to this accounting problem but are not appropriate for biodiversity studies where the goal is to discover novel taxa. Inconsistent taxonomic assignments for reference sequences in public databases further challenge comparative molecular descriptive studies. Despite these obstacles, we have learned much about the phylogenetic diversity of microbial eukaryotes using culture-independent approaches.

This study compared eukaryotic communities from naturally occurring and anthropogenic sites with pH values from 2 to 11 in diverse aquatic environments ranging from ARDs of the Rio Tinto, Spain to the alkaline hydrothermal vent fields of Lost City using massively-parallel pyrotag sequencing targeting the V9 hypervariable region of the 18S rRNA gene. The aim of this study was to determine what groups of eukaryotic taxa typify extremely acidic or extremely alkaline environments and how cosmopolitan these groups are at pH extremes. This was tested using a combination of hierarchical clustering, indicator species analysis (ISA), non-metric multidimensional scaling (NMDS), and oligotyping analyses. The advent of next-generation sequencing and its application to biodiversity studies in extreme environments is broadening our understanding of the limits to life on our planet and will aid in determining which groups of taxa are best suited to become model organisms to increase our understanding of adaptation to life's extremes.

## Methods

### Site characteristics

**Figure 1:**
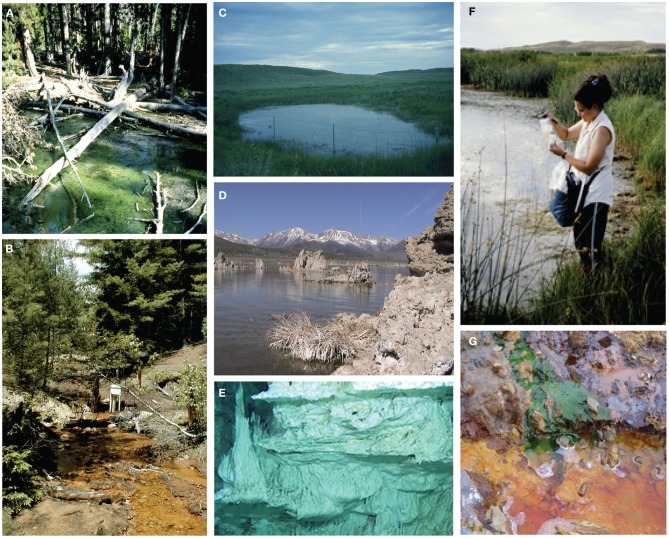
**pH extreme sites examined in this study. (A)** Nymph Creek, Yellowstone National Park (YNP), WY, USA. **(B)** Davis Mine, Rowe, MA, USA. **(C)** Sandhills Lake Between Tree Claim and Smith, Crescent Lake Wildlife Refuge, NE, USA. **(D)** South Tufa at Mono Lake, CA, USA. **(E)** Lost City Hydrothermal Vent Field, Mid-Atlantic Ridge (photo credit, IFE, URI—IAO, UW, Lost City science party, and NOAA). **(F)** Mallard Lake, Crescent Lake Wildlife Refuge, NE, USA. **(G)** Anabel's Garden, Rio Tinto, Nerva, Spain. See text for details of site descriptions.

**Figure 2:**
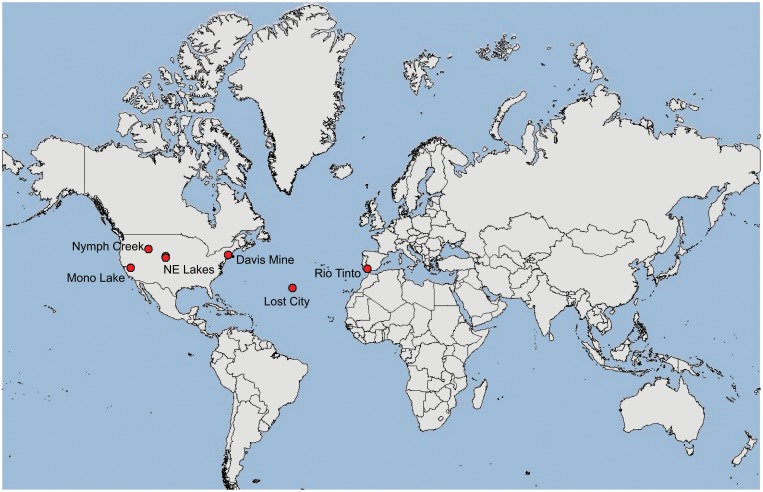
**Map of sampling site locations**.

The pH extreme environments examined in this study ranged from extremely acidic (pH 2) to extremely alkaline (pH 11) (Figures 1A–G, Table 1). The extreme acidic sites included Nymph Creek, YNP, WY, Davis Mine in Rowe, MA, and Anabel's Garden in the Rio Tinto in southwestern Spain, while our extreme alkaline sites included two Sandhills region lakes in Nebraska, Mono Lake in California, and the Lost City Hydrothermal Vent on the Mid-Atlantic Ridge in the Atlantic Ocean. Nymph Creek (Figure 1A) is located near the Norris Geyser basin in YNP, WY and is characterized as a ferrous iron (2.3 mg/L) and arsenite-rich (0.086 mg/L) shallow thermal spring with temperatures ranging from 38°C to 52°C (Nordstrom et al., 2005). Its most salient feature is the green microbial mat that blankets the bottom of the creek composed of red and green algal species (Brock, 1978). Davis Mine (Figure 1B) in Rowe, MA, is the former site of the Davis Sulphur Ore Company that operated during the late 1800's to provide a commercial source of sulfuric acid (McCarthy, 1977). The site now contains AMD streams of high iron content. Anabel's Garden (Figure 1G) in the Rio Tinto, Spain is the site of an extensively studied ARD area that is situated on the Iberian Pyritic belt, the largest metal sulfide surface deposit in the world. Abundant and distinct biofilms of *Euglena* and diatoms typify this site fed by a small stream and groundwater derived from a lake in an abandoned mining pit. Alkaline lakes in the Sandhills region of NE are located in the Crescent Lake National Wildlife Refuge of Garden County (Figures 1C,F). Two of these lakes were included in this study and are typical of alkaline lakes in this region. While no direct physico-chemical measurements other than pH accompanied the sampling of the alkaline lakes, Sandhills lakes in general are classified as sodium and potassium-bicarbonate-carbonate-hydroxide varieties where sodium, potassium, calcium, and magnesium are the most numerous cations found (McCarraher, 1977). Mono Lake (Figure 1D) in California served as an alkaline, hypersaline site characterized by dominance of carbonate, sulfate and sodium ions. Mono Lake contains elevated dissolved organic carbon (DOC) and high bacterial concentrations (Hollibaugh et al., 2001). The final alkaline site, Lost City (Figure 1E) is an ancient hydrothermal vent system rich in calcium but low in heavy metals with abiogenic sources of hydrogen, as well as methane and hydrocarbons (Proskurowski et al., 2008). Table 1 lists the origin and associated contextual data for all samples and Figure 2 shows the geographic locations of each site.

**Table 1:** **Metadata and contextual data associated with the samples used in this study**.

**Sample name**	**Sampling description/location**	**Collection date**	**Sample type**	**pH**	**Temperatures (°C)**	**Reads**	**Ave. length (nt)**	**OTU_obs_**
RT7II	Anabel's Garden, Rio Tinto, Nerva, Spain	18 April 1999	Biofilm/sediment	2.0	18.5	17,077	126	32
DM2	Davis Mine, Rowe MA, USA	19 December 1998	Biofilm/sediment	2.7	na	4521	130	84
WY1	Nymph Creek, Yellowstone, WY, USA	23 July 1999	Biofilm/sediment	2.75	40	9432	124	92
ML1	South Tufa, Mono Lake, CA USA	25 May 2003	Water/biofilm	9.7	21.6	14,576	129	56
NE2	Between Tree Claim and Smith, Sandhills NE, USA	18 July 1999	Biofilm/sediment	10.3	28.6	20,557	132	265
NE3	Mallard Lake, Sandhills NE, USA	18 July 1999	Biofilm/sediment	10.4	33.1	11,990	134	208
LCY	Lost City, Mid-Atlantic Ridge	April/May 2003	Chimney/biofilm	11	7–88	4214	124	173

Number of reads refers to the number of quality-checked reads and the OTU_obs_ is the observed richness after random resampling the datasets down to the lowest sampling effort (4214 reads). Ave. length refers to the average length of amplicon reads for a sample.

### DNA extraction

Genomic DNA extraction methods varied and followed protocols optimized for a given extreme environment. Sediment samples were collected aseptically using a 50-ml centrifuge tube and immediately frozen in a liquid nitrogen dry shipper, then transferred to a −80°C freezer upon return to the laboratory. The Mono Lake water sample containing resuspended biofilm material (500 ml) was filtered onto a 0.2 μm Sterivex filter (Millipore Corp., Beverley, MA) and extracted using the Puregene Tissue kit (Qiagen, Valencia, CA) and methods. Biofilm/sediment extractions (1 g each) followed a modified phenol-CTAB extraction procedure (Hugo et al., 1992). The biofilm/sediment sample from the Rio Tinto (RT7II) was extracted as described in Amaral-Zettler et al. (2002) and Lost City chimney samples were extracted according to protocols in Brazelton et al. (2010). The Lost City sample was a physical pool of four separate samples so represents a range of temperatures as indicated in Table 1.

### Pyrosequencing and data analysis

Pyrosequencing of all samples followed protocols established in Amaral-Zettler et al. (2009) and applied barcoded primers to achieve multiplexing of samples on the Genome Sequencer FLX Platform (Roche, Basel, Switzerland) housed in the Marine Biological Laboratory Keck Sequencing Facility and used the GS-LR70 long-read sequencing kit (Roche). The tag recovery and read length for all samples varied and is summarized in Table 1. Sequences were trimmed and screened for quality after Huse et al. (2007). To assign taxonomy to the remaining quality-controlled tags, we used the global alignment for sequence taxonomy (GAST) algorithm (Huse et al., 2008) with version 111 of the Silva-ARB database (Pruesse et al., 2007) that has recently been updated with improved eukaryotic taxonomic assignments. V9 amplicon reads were grouped into operational taxonomic units (OTUs) using SLP-PWAN (Huse et al., 2010) at 6% cluster widths. The open source investigation/study/assay (Sansone et al., 2012) metadata-tracking framework was used to curate the datasets and format them for submission to the NCBI sequence read archive (SRA) database under the SRA number SRA059386. All data are MIMARKS compliant (Yilmaz et al., 2011).

Tools available in R (www.r-project.org) helped to calculate Venn diagrams constructed from the full dataset that were visualized using the Venn Diagram Plotter (http://ncrr.pnl.gov/ or http://www.sysbio.org/resources/staff/). For the purposes of drawing the Venn diagrams alone, the OTU data for the Sandhills lakes were pooled so as to represent the overlapping communities in a three-way Venn diagram. All multivariate analyses were performed on a randomly resampled data matrix to standardize for sampling effort (4214 reads). The matrix was subsequently transformed into incidence (presence/absence) data. Hierarchical clustering and similarity profile (SIMPROF) permutation tests were implemented in Primer E (Clarke and Warwich, 2001) and used Jaccard distances. Similarity percentage (SIMPER) analysis, also implemented in Primer E, identified the OTUs that contributed the most to the separation between the acidic and alkaline groups. NMDS (Mather, 1976) was implemented in the PC-ORD software (Peck, 2010). Replicate stress tests allowed for selection of the optimal dimensionality for the NMDS. The final NMDS solution employed a Jaccard distance measure, used the local computer time to generate a random start seed, and included 250 runs with real data and 250 runs with randomized data. A 3-dimension final solution with 24 iterations was deemed optimal with a *P* value of 0.0199 using a Monte Carlo test. The final stress and final instability were 0 and 0.00053, respectively.

ISA (Peck, 2010) helped to test the hypothesis that different extremophile OTUs define extremely acidic and extremely alkaline environments based on constancy and abundance in a given group. Since the V9 region of 18S rRNA genes is not typically able to differentiate between OTUs at the species level it is more appropriate to refer to this analysis as indicator OTU analysis (IOA). Two groups were chosen on the basis of pH as determined by the hierarchical clustering results: samples falling into the extreme acidic (0–3) pH group and those falling into the extreme alkaline (9–11) pH group. This method can involve either abundance or presence/absence matrices. Randomly resampled (4214 reads for each sample) abundance and binary matrices were employed for this analysis as appropriate. IOA was performed using the Dufrêne and Legendre (1997) method to obtain an “indicator value” (IV), the product of relative abundance and constancy that ranges from 0 to 100. A second method employed the Tichý and Chytrý approach (2006) wherein IV values are reported as a perfect negative indication (−1) or a perfect positive indication (1). Random reassignment of the samples to groups followed by iteration of the IV calculation provided a test of the statistical significance of the IV values.

The R package routines gplots and heatmap.2 were used to generate the heatmap summary of all OTUs that were encountered with a frequency of greater than 1% in a given site. An oligotyping analysis (Eren et al., 2011) was performed on the two OTUs identified to the genus level identified using IOA. Oligotyping involved retrieving all reads that fell into the E19 (assigned the genus *Pinnularia*) and E112 (assigned to the genus *Frontonia*) 6% OTUs clusters, respectively. Sequence reads were then aligned in MUSCLE (Edgar, 2004) to obtain a multiple sequence alignment. Shannon entropy analysis was performed on the aligned OTU reads to quantify nucleotide uncertainty along the columns of aligned sequences and identify highly variable nucleotide positions. Oligotyping then decomposed the data to identify meaningful groups within the samples examined. The power in this technique is that it can identify evolutionary units missed by distance-based approaches commonly employed in microbial community analyses.

## Results and discussion

### Alpha diversity

Alpha diversity, the number of OTUs (OTU richness) within a given site, is a metric often employed in biodiversity studies to contrast communities from different environments. It is generally accepted that harsh environments experiencing one or more stressors tend to harbor fewer species (Frontier, 1985). This study is the first to compare and contrast eukaryotic diversity in extreme environments spanning 9 orders of magnitude in hydrogen ion concentration. A total of 946 OTUs were recovered at a 6% cut-off level (94% similarity) across seven sampled pH extreme environments. Clustering at this conservative cut-off helped to compensate for possible intraspecific heterogeneities in rRNA gene copies (Amaral Zettler et al., 1998), and the SLP-PWAN approach with 2% single linkage preclustering followed by 6% average neighbor clustering further minimized OTU inflation that can occur from pyrosequenced-generated homopolymers (Huse et al., 2010; Quince et al., 2011). Only observed and not estimated richness values were calculated on these datasets because replicated samples were not available to perform incidence-based diversity estimation better suited to taxa with highly variable marker gene copy number. Although sequenced amplicon read recoveries varied from 4214 to 20,557, resampling to normalize for sampling effort revealed richness values (recorded as observed OTUs) wherein the alkaline samples (e.g., Between Tree-Claim and Smith) were the most OTU-rich while acidic ones (e.g., Anabel's Garden, Rio Tinto) were the least (Table 1).

Within acidic sites, the Rio Tinto yielded the fewest observed numbers of OTUs (32) while Nymph Creek revealed the largest (92). In contrast, alkaline sites were much more OTU-rich, on average yielding several times greater OTU richness than their acidic counterparts. The exception was Mono Lake, where only 56 OTUs were recovered after normalizing for sampling effort. If one associates decreased richness with greater stress in a given environment, then these results support the hypothesis of overall lower diversity at increased environmental extremes. For example, Mono Lake possesses high pH, high levels of certain toxic metals (e.g., arsenic) and is hypersaline. Similarly, in the Rio Tinto, a combination of low pH and high toxic metal concentrations both likely contribute to habitat filtering excluding the existence of a more phylogenetically diverse assemblage of organisms (Amaral-Zettler et al., 2011). Like the Rio Tinto and Mono Lake, where both extreme pH and high metals prevail, the Lost City site extremes are multi-faceted and include high pH, temperature and pressure extremes. Surprisingly, Lost City still yielded relatively higher observed OTU-richness despite the added extremes encountered. One possible explanation may have been that the comparatively reduced metal concentrations characteristic of the Lost City geochemistry (Brazelton et al., 2010) reduces the effects of habitat filtering in this environment. Another important consideration in contrasting richness patterns using any PCR-based approach is the contribution of DNA derived from allochthonous sources (e.g., surrounding seawater) and environmental DNA from inactive or dead cells (Pawlowski et al., 2011). It is well-known that DNA is more chemically stable under alkaline than acidic conditions and thus one might expect that extremely acidic environments would be less likely to contain large amounts of persistent DNA from inactive organisms or foreign sources (Bernhardt and Tate, 2012) while alkaline environments might reflect the opposite trend. Work by Lopez-Garcia et al. (2003) on acidic hydrothermal vents suggested only autochthonous communities were detected based on an absence of photosynthetic taxa. The application of next-generation sequencing to environmental biodiversity surveys, however, is a much more sensitive technique and thus data resulting from next-generation approaches need to be interpreted with caution even in environments seemingly inhospitable to DNA preservation outside the cell.

### Beta diversity and community composition

**Figure 3:**
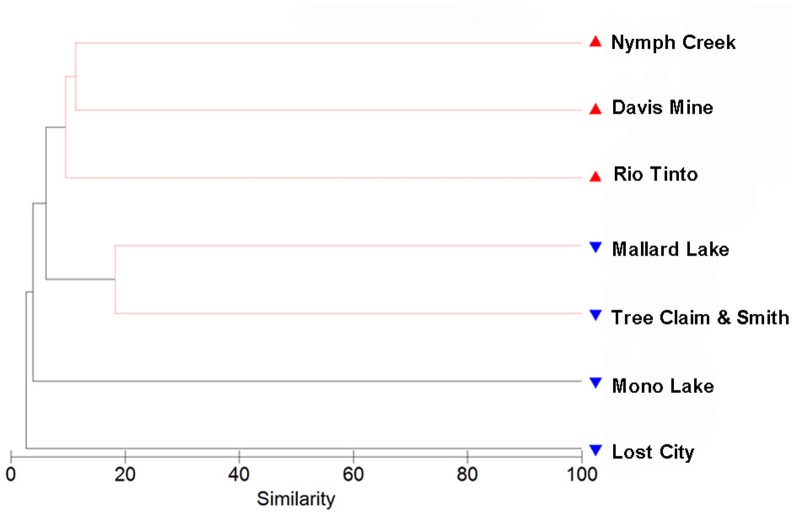
**Hierarchical clustering analysis using a resemblance matrix composed of Jaccard derived similarities between samples.** Acidic samples are indicated with red symbols while alkaline samples are indicated with blue symbols. A similarity profile (SIMPROF) permutation test highlights in red clusters that show significant internal structure.

**Figure 4:**
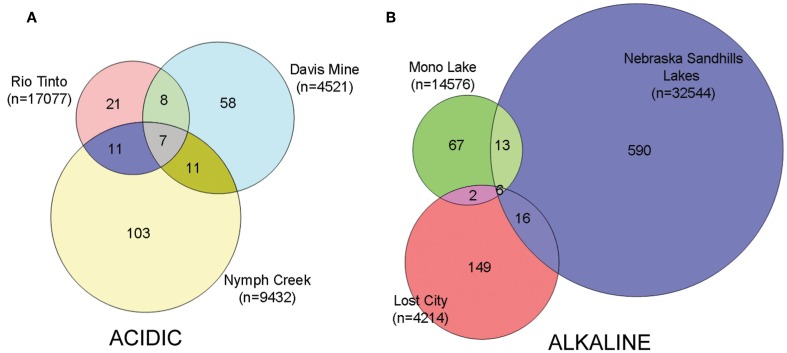
**Venn diagrams indicating the degree of overlap between (A) acidic OTUs and (B) alkaline OTUs.** Total number of amplicon reads per sample is indicated in parentheses under the sample names. Nebraska Sandhills samples were pooled only for the purposes of drawing the Venn diagram. Non-resampled data sets were used to generate these diagrams.

Hierarchical clustering of the sites separated acidic from alkaline communities with greatest support for the clustering of the three acidic sites and the two Nebraskan Sandhills Lake sites (Figure 3). Despite the segregation of the acidic sites from alkaline sites, overall similarities between sites were low. All sites had large proportions of unique OTUs but shared OTUs with other sites as well (Figure 4). For example, Rio Tinto's acidic Anabel's Garden contained ~45% unique OTUs and shared close to a third of its OTUs with either Davis Mine or Nymph Creek. Alkaline sites had proportionally fewer shared OTUs with ~76% to ~94% unique OTUs (when OTUs from the Sandhills Lake sites were pooled). A SIMPER analysis highlighted the shared OTUs within acidic and alkaline sites, respectively (Tables A1 and A2). The average similarity between the acidic sites was 18.48% while that for the alkaline sites was 9.92%. The most cosmopolitan OTU was a diatom that occurred across all seven sites. This OTU (E81) was assigned to the diatom class Fragillariophyceae with an exact match over the entire V9 region to several different diatom genera including three different species of *Diatoma*, and a single species each of *Staurosira*, *Tabellaria*, and *Asterionella*. Although the level of resolution attainable using the eukaryotic V9 hypervariable region does not readily differentiate between species (and in this example, genera), some authors argue that, with a few exceptions, most diatom genera are cosmopolitan (Vanormelingen et al., 2008). It is noteworthy that members of the genus *Diatoma* in particular have been reported from alkaline (Reavie and Smol, 2001) and acidic (Denicola, 2000) environments. What is less certain is whether a given species is capable of surviving extremes captured by the sites examined in this study (but see, Costas et al., 2007). However, at least some algal species such as *Picocystis* sp. are known to be capable of existing at pH values ranging from 4 to 12 (Roesler et al., 2002) despite a preference for alkaline pH, indicating that the ability to adapt to pH over broad ranges does exist in some evolutionary lineages.

**Figure 5:**
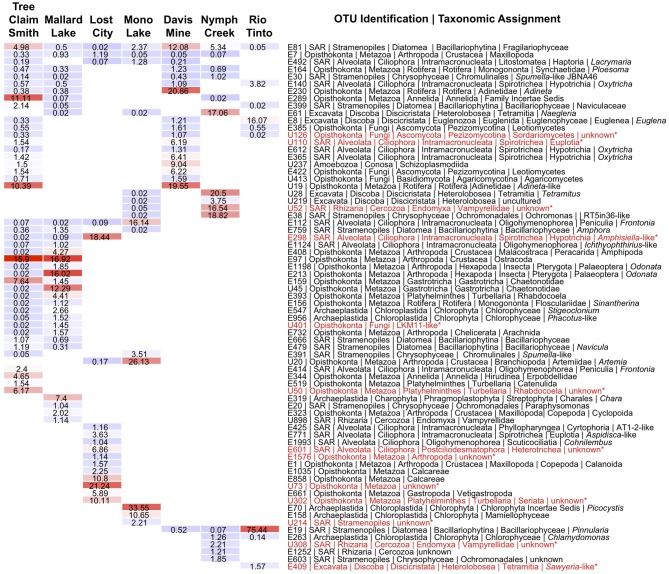
**Heatmap of OTUs occurring at a frequency of more than 1% at a given site.** Alkaline sites are the four on the left and acidic sites the three on the right and are arranged in order of decreasing OTU richness from left to right. OTUs that occur at both alkaline and acidic sites are arranged at the top of the table from most to least cosmopolitan, while OTUs occurring at only alkaline or acidic sites are arranged below. Hot colors indicate higher percentages while cooler colors indicate lower percentages, with actual values superimposed over colors. Highlighted in red and with an asterisk are OTUs that had either GAST or BLAST distances of greater than 9% to any known sequence in GenBank. Data shown are normalized by randomly resampling data to the lowest sampling effort. The depth of taxonomic resolution available in the V9 region varied by taxon and is displayed to the right.

Apart from the afore-mentioned diatom E81 OTU, a total of 5 additional OTUs were shared across all alkaline sites while 6 were shared across acidic ones. Among the shared alkaline OTUs were ciliates assigned to members of the genera *Frontonia* and *Lacrymaria*, a cercozoan OTU assigned to the genus *Protaspa*, a maxillopod metazoan OTU and a tracheophyte OTU. Shared acidic OTUs included those assigned to the diatom genus *Pinnularia*, a chrysophyte OTU, and opisthokont OTUs including fungi, a holozoan and a maxillopod. Figure 5 illustrates the OTUs found in the seven sites at a frequency greater than 1%. Some of these OTUs were found exclusively at a given site and were the dominant OTU at that site. Examples included an unidentified opisthokont OTU from Lost City with a top BLAST hit of only 90% similarity to a gammarid amphipod and the unicellular chlorophyte *Picocystis* from Mono Lake. *Picocystis* is well-documented in the Mono Lake ecosystem where it accounts for up to 50% of the primary production there at certain times of the year (Roesler et al., 2002).

The heatmap (Figure 5) also highlights the relative dominance of the diatom OTU E19 assigned to the genus *Pinnularia* at the Rio Tinto site, which unlike the E81 diatom OTU, could be definitively assigned to this genus. *Pinnularia* is a known acidophilic diatom genus that includes several species that have been reported from both AMD environments (Luis et al., 2012) and hot springs in Yellowstone National Park (Denicola, 2000). Previous work in the Rio Tinto using a polyphasic approach combining molecular and morphological approaches has also shown *Pinnularia* to be the dominant diatom at this site (Souza-Egipsy et al., 2008).

**Figure 6:**
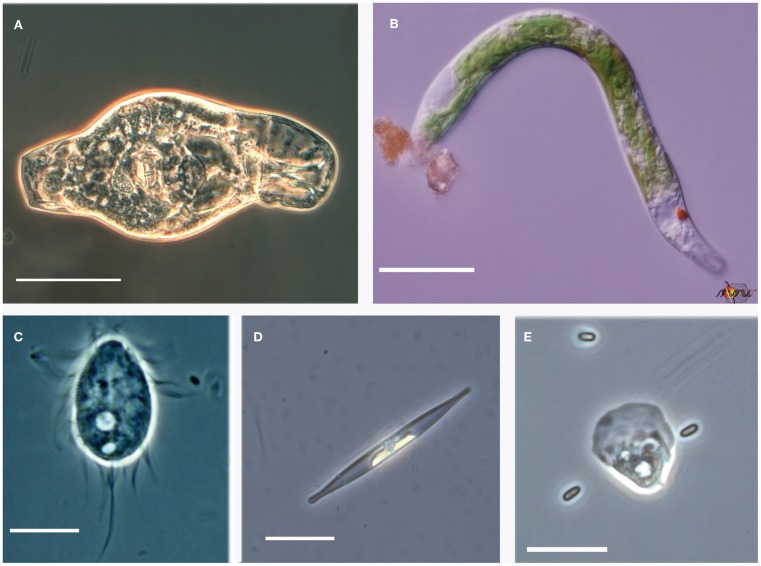
**Examples of pH extremophiles. (A)** Rotifer from the Rio Tinto (scale bar = 50 μm). **(B)** *Euglena mutabilis* from Davis Mine, MA (scale bar = 20 μm). **(C)** *Cyclidium* sp. ciliate from Mallard Lake, NE (scale bar = 10 μm). **(D)** Mono Lake diatom from South Tufa (scale bar = 20 μm). **(E)** Lobose amoeba with yeast from Rio Tinto (scale bar = 20 μm).

As a general trend, distinct OTUs numerically dominated at different pH extreme sites (Figure 5) regardless of similarity in pH or geographic proximity. Metazoan OTUs were well represented among them. At Davis Mine for example, rotifer OTUs related to the bdelloid genus *Adineta* encompassed the largest number of reads while in both of the Nebraskan alkaline lakes ostracods were the dominant OTU. In addition to ostracods, additional metazoan OTUs in the Sandhills Lakes included annelid, gastrotrich, amphipod, rotifer, and insect larvae (*Odonata*). The broad range of metazoa that characterized these sites likely indicates that these particular alkaline environments are more hospitable for multicellular life than the other extreme environments examined in this study. However, some metazoa such as rotifers, are known to thrive at a range of pH extremes including high metal environments (Horvath and Hummon, 1980; Deneke, 2000; Bell, 2012) and this observation was confirmed in this study. While no rotifer OTUs were detected in the sample examined from the Rio Tinto, other sites along this river do contain rotifers (Figure 6A, personal observation) but typically at pH values closer to 3. Metazoan OTUs likewise dominated the Lost City hydrothermal vent site. At Lost City, apart from the potentially novel (metazoan) opisthokont OTU, also common was a calcareous sponge OTU that is likely well-adapted to the calcium-carbonate-rich chemistry of the vent. At other sites protistan OTUs dominated. A dominant ciliate OTU at Lost City was only found at alkaline sites, was not closely related to other sequences recovered in previous molecular surveys of Lost City (López-García et al., 2007), and had an overall low BLAST similarity assignment to sequences in GenBank (91%), indicating it may be quite novel. In contrast, other ciliate OTUs detected in the Lost City site including OTUs assigned to Lost City ciliate clone AT1-2 and the genus *Cohnilembus* appear to be related to ones detected by López-García and colleagues.

The most abundant OTU at Nymph Creek was 98% identical to the heterolobosean amoeba *Tetramitus thermoacidophilus*—an amoeboflagellate isolated from acidic hot springs in Italy and Kamchatka (Baumgartner et al., 2009). The Italian site where this amoeba was isolated was similar to Nymph Creek in being covered with *Cyanidium*-like algae and possessing a pH of 3, but was warmer in temperature at 72°C. The amoeba was described as a benthic thermo-acidophile with optimal growth at low pH and elevated temperatures. This same OTU was rare but also found in Mono Lake so acidity and high temperature may not constrain its distribution. The second most abundant Nymph Creek OTU was a flagellate identical to a clone from Rio Tinto [RT5in36; (Amaral Zettler et al., 2002)], but did not occur at Anabel's Garden examined in this study. Representative sequences from the third most abundant Nymph Creek OTU matched various species of the heterolobosean amoeba *Naegleria* with 98% similarity including *Naegleria*. sp. COHH, *N. gruberi*, *N. australiensis*, and others. Members of this genus include human pathogenic species reported from other YNP hot springs (Sheehan et al., 2003) and are thus of interest from the human health perspective. The potentially novel vampyrellid OTUs (U308 and U52) may be effective predators on the overlying algal biofilm biomass in Nymph Creek given that many representatives of this group are known to selectively feed on algae as prey (Hess et al., 2012).

The occurrence of similar taxa across seemingly physico-chemically distinct environments calls into the question the relative influence pH imparts on the community structure of eukaryotes living at pH extremes. While most abundant OTUs were predictable, there were exceptions. The presence of the ubiquitous E81 diatom OTU must be interpreted with caution due to the limits of taxonomic resolution afforded by the V9 region for this OTU. Another curious observation, however, was an absence of *Cyanidium* sequences in the Nymph Creek sample and the low frequency with which *Chlorella*-like OTUs were detected (<1% frequency) there. These taxa have been reported as dominant in this system, but the absence of *Cyanidium* in Nymph Creek may corroborate the findings of Ferris et al. (2005) who noted a shift from *Cyanidium* to *Chlorella* species in the cooler sections of the Creek. However, the lack of dominance of either *Cyanidium* to *Chlorella* is surprising. It may also be possible that extraction methods employed in this study were insufficient to recover larger proportions of *Cyanidium* or *Chlorella*-like OTUs from the samples given that these cells are sometimes difficult to lyse. An alternative explanation for the low algal recoveries may be low copy numbers of rRNA genes in these taxa relative to those of the amoebae that dominated the diversity at this site.

Diatom and euglenid OTUs dominated the Rio Tinto site. Both *Pinnularia* and *Euglena mutabilis* are “flagship” taxa known to occur in the conspicuous biofilms at this site (Amaral Zettler et al., 2002; Aguilera et al., 2007) and have such distinct morphologies that they allow unambiguous identification (Foissner, 2006) in the absence of molecular ecological approaches. The detection of both the *Pinnularia* and *Euglena* OTUs in this study is noteworthy, however, because this same sample was examined during the first descriptions of biofilm diversity in the Rio Tinto (Amaral Zettler et al., 2002) using a clone-library-based approach targeting full-length rRNA genes and did not recover the *Euglena* OTU. The difference between this study and the previous one was that this study targeted the much shorter and less length variable V9 hypervariable region of the 18S rRNA gene. Length variations rRNA genes are known to cause systematic amplification biases wherein shorter regions are preferentially amplified over longer ones (Huber et al., 2009). The length of the *E. mutabilis* 18S rRNA gene is ~2500 bp, nearly 700 bp longer than typical eukaryotic rRNA genes and was biased against in earlier work. A similar problem was encountered in a study at the same Rio Tinto Anabel's Garden site sampled during a different season using 3-domain specific primers targeting the V4–V8 region (Amaral-Zettler et al., 2011). In the latter study, only the shorter chloroplast 16S rRNA genes from *E. mutabilis* were recovered and not the longer nuclear versions. Therefore, for the purposes of biodiversity discovery, there are still advantages to targeting shorter regions such as the V9 that, in addition to being less prone to chimera-formation, are also less prone to amplification length biases.

One of the advantages of deep sequencing is the recovery of both abundant and rare microbial community members (Sogin et al., 2006). The detection of rare taxa also invites the discovery of novel taxa as well. Alongside the more conspicuous Rio Tinto photosynthetic biofilm community members already mentioned were other OTUs that constituted lesser readily observed heterotrophic members of the community including a ciliate OTU assigned to the genus *Oxytricha*, a *Sawyeria*-like heterolobosean amoeba OTU, and fungal OTUs related to those previously characterized in the Gadanho et al. (2006) study. The concordance of the most abundant OTUs found in the Rio Tinto with previous studies and the detection of a potentially novel fungal OTU (U126) occurring at another ARD site with greater than 1% representation lend support to the value of the comparative approach taken in this study. Figure 6 provides additional examples of some of the common acidophiles and alkaliphiles observed by microscopy during this study.

**Figure A1:**
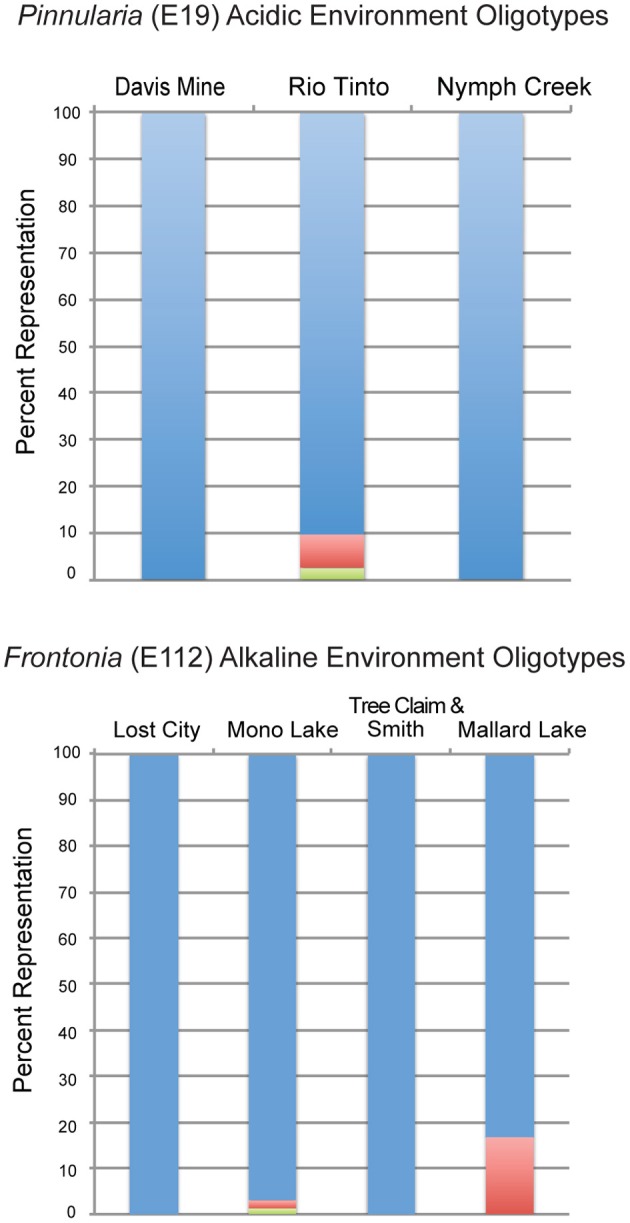
**Oligotypes detected in indicator OTUs identified to the genus level.** Different colors represent different oligotypes.

A search for taxa indicative of acidic or alkaline extremes is summarized in the IOA analysis (Table A3). These results indicated that *Pinnularia* (E19) and two different opisthokont OTUs of holozoan (E928) and fungal (U2611) affiliation were significant indicator OTUs of acidic environments. Species within the genus *Pinnularia* have been described as having the ability to disperse globally with even isolates derived from neutral pH environments being capable of acid-tolerance (Ciniglia et al., 2007). There was only one indicator taxon among the alkaline sites examined, likely because these sites were so geochemically diverse and thus hosted relatively distinct communities. In the IOA analyses, a single OTU (E112) assigned to the ciliate genus *Frontonia* was identified as a strong candidate for indicating extreme alkaline conditions using both abundance-based and presence/absence-based IOA methods (only Dufrêne and Legendre abundance data are shown). Published reports of *Frontonia* from saline alkaline environments exist from Kenya (Yasindi et al., 2002) and sequences related to *Frontonia* were detected in the López-García et al. Lost City study (2007). In the former study, *Frontonia* was determined to contribute up to 58% of the ciliate production in the lake. Due to their large size and potential to ingest larger prey, these ciliates may serve the role of top predator in extreme environments that don't support a large metazoan population. However, ciliate size does not necessarily correlate with their predatory role. While this study did not attempt to examine indicators at the “species” level, closer examination of the *Pinnularia* and *Frontonia* OTUs using an oligotyping approach still revealed one predominant oligotype shared across all the acidic or all the alkaline sites, respectively (Figure A1). For the *Pinnularia* OTU cluster, two additional oligotypes were detected in the Rio Tinto sample while for the *Frontonia* OTU cluster, two additional oligotypes were detected with one being shared by Mono Lake and Mallard Lake and the second found only in Mono Lake.

The IOA identified statistically significant pH indicators within acidic and alkaline groups of sites but insights can also be gained from examining shared OTUs across sites. For example, although not the major focus of this study, it has long been recognized that rotifer distributions are linked to pH (Edmondson, 1944) where they have been subsequently used in water quality studies (Horvath and Hummon, 1980; Siegfried et al., 1989). In this study we found that *Adineta* and *Adineta*-like OTUs (E230 and U19) occurred at both the acidic Davis Mine, as well as one or more of the alkaline Sandhills Lakes. The occurrence of rotifers over a broad range of pH values is well documented and a study in Sweden reported *Adineta vaga* with a range of pH from 3 to 10 (Berzins and Pejler, 1987), similar to the pH difference measured at sites in this study. The genus *Ploesoma* also detected in this study often occurs in humic and acidic waters that may explain its distribution at both acidic and alkaline sites where it may be associated with leaf litter. Other rotifer OTUs such as E156 assigned to the genus *Sinantherina* were only found in the Sandhills alkaline lakes and members of this genus are known to have species restricted to alkaline waters (Smith, 2001). There is less information on the biogeography of microbial eukaryotes than rotifers with respect to pH environments so it is difficult to say whether a given microbial eukaryotic species possesses broad pH tolerance. While the ability for microbial eukaryotes to adapt to pH extremes over a broad range of values may be rare, this study has revealed that diatoms appear to be good candidate taxa that may be able to span this gamut. Other possible candidates to examine more closely include representative ciliate (e.g., *Oxytricha*), flagellate (e.g., *Spumella*), and amoeboid (e.g., Heterolobosean) protists.

**Figure 7:**
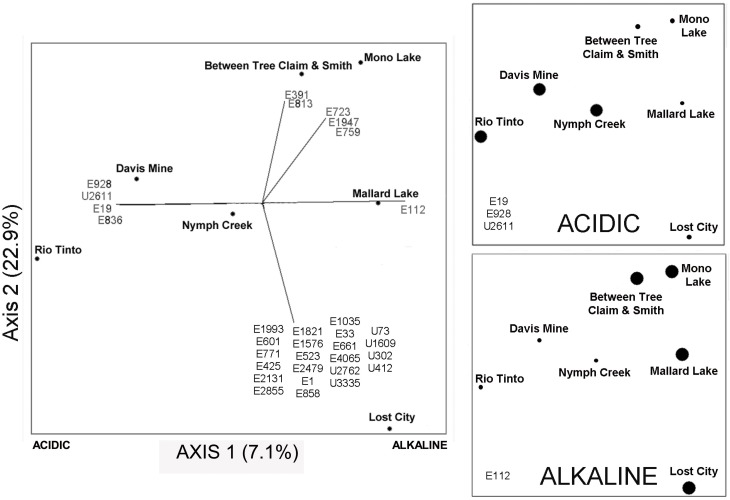
**NMDS plot on the left with Jaccard distances showing the proportion of variance contributed by each axis (calculated from the *r*^2^ between distance in the ordination space and in the original space).** Small circles on the left-hand plot show the location of all samples in the ordination. The two panels on the right show an overlay in the same ordination space of the “indicator” OTUs that are found in all acidic samples, upper panel (Rio Tinto, Davis Mine, and Nymph Creek), and in the lower panel the OTU found in all alkaline samples (Between Tree Claim and Smith, Mallard Lake, Mono Lake, and Lost City). Large circles in the two right-hand panels indicate the presence of numbered indicator OTUs in the lower left-hand corners of these plots, while small circles indicate their absence. See Table A4 for taxonomic assignments of representative OTU numbers shown on all plots.

The goal of this study was to compare eukaryotic diversity across a range of pH extremes. The distinct sites described here yielded ordinations that separated pH extreme sites but this was only able to explain a modest fraction in the variation of the data (7.1%; Figure 7). Other factors in addition to pH clearly influence the underlying community structure in these environments. Even though pH appears to be a dominant factor structuring bacterial communities in soils (Fierer and Jackson, 2006), other factors such as salinity heavily influences the underlying community structure in aquatic environments (Lozupone and Knight, 2007). In the case of the sites examined in this study that include ranges of salinity and metal ion concentrations, a likely factor shaping community structure is metal ion concentrations, many of which are known to be toxic to microbes and macrobes alike.

## Conclusions

Comparative molecular ecology studies at pH extremes allow us to identify taxa that may be broadly adapted to both acidic and alkaline pH and thus able to more readily attain cosmopolitan distributions. Many of the OTUs identified in this study were assigned to genera that are capable of widespread passive dispersal via resting or vegetative cysts or active dispersal via animal or human vectors. Active dispersal via ingestion exposes organisms to a broad range of pH within the gut of a given vector and thus tolerance to a wide range of pH values may impart a competitive advantage. Alkaline sites exhibited higher alpha diversity (larger number of observed OTUs) versus acidic sites overall and high metal-containing sites were the least rich within acidic and alkaline groups, respectively. Beta diversity analyses indicated low similarity between and within acidic and alkaline environments with few shared OTUs even with a conservative clustering OTU cut-off of 6%. IOA suggested that the diatom genus *Pinnularia* and the ciliate genus *Frontonia* are good indicators of acidic and alkaline conditions, respectively, but the taxonomic resolution achievable via examination of the V9 18S rRNA hypervariable region alone does not allow for discrimination at the species level. Despite this limitation, the V9 region does allow for a relatively unbiased first look comparing eukaryotic diversity in underexplored habitats such as pH extreme environments. Future studies targeting cosmopolitan pH extreme taxa using a battery of additional marker genes (Pawlowski et al., 2012) may provide important insights into the ecology, biogeography, and diversity of eukaryotes at pH extremes.

### Conflict of interest statement

The author declares that the research was conducted in the absence of any commercial or financial relationships that could be construed as a potential conflict of interest.

## Appendix

**Table A1:** **Shared OTUs from acidic sites determined by SIMPER analysis**.

**OTU ID**	**Ave. abun**	**Ave. sim**	**Sim/SD**	**Contr. %**	**Cum. %**	**Taxonomy**
E19	1	1.49	4.78	8.07	8.07	Stramenopiles; Diatomea; Bacillariophytina; Bacillariophyceae; *Pinnularia*
E81	1	1.49	4.78	8.07	16.14	Stramenopiles; Diatomea; Bacillaryophytina; Fragillariophyceae
E928	1	1.49	4.78	8.07	24.21	Opisthokonta; Holozoa; Filasterea
U2611	1	1.49	4.78	8.07	32.28	Opisthokonta; Fungi; environmental samples
E8	0.67	0.57	0.58	3.11	35.39	Excavata; Discoba; Discicristata; Euglenozoa; Euglenophyceae; *Euglena*
E140	0.67	0.57	0.58	3.11	38.5	Alveolata; Ciliophora; Intramacronucleata; Spirotrichea; Hypotrichia; *Oxytricha*
E385	0.67	0.57	0.58	3.11	41.61	Opisthokonta; Fungi; Ascomycota; Pezizomycotina; Leotiomycetes
U126	0.67	0.57	0.58	3.11	44.72	Opisthokonta; Fungi; Ascomycota; Pezizomycotina; Sordariomycetes; unknown
E836	0.67	0.57	0.58	3.11	47.83	Opisthokonta; Fungi; Nucletmycea; *Nuclearia*
E47	0.67	0.57	0.58	3.11	50.94	Discoba; Discicristata; Euglenozoa; Kinetoplastea; *Eubodonida*
E263	0.67	0.54	0.58	2.91	53.85	Archaeplastida; Chloroplastida; Chlorophyta; Chlorophyceae; *Chlamydomonas*
E818	0.67	0.54	0.58	2.91	56.76	Archaeplastida; Chloroplastida; Chlorophyta; Trebouxiophyceae; *Nannochloris*
U105	0.67	0.54	0.58	2.91	59.67	Archaeplastida; Chloroplastida; Chlorophyta; Chlorophyceae; *Chloromonas*
U22	0.67	0.54	0.58	2.91	62.57	Rhizaria; Cercozoa; Thecofilosea
E3	0.67	0.54	0.58	2.91	65.48	Alveolata; Dinoflagellata; Dinophyceae
E137	0.67	0.54	0.58	2.91	68.39	Excavata; Discoba; Discicristata; Euglenozoa; Kinetoplastea; Trypanosomatida
E157	0.67	0.54	0.58	2.91	71.3	Alveolata; Ciliophora; Intramacronucleata; Litostomatea; Haptoria; *Balantidion*
E164	0.67	0.38	0.58	2.05	73.35	Opisthokonta; Metazoa; Rotifera; Monogononta; Synchaetidae; *Ploesoma*
E7	0.67	0.38	0.58	2.05	75.4	Opisthokonta; Metazoa; Arthropoda; Crustacea; Maxillopoda
E30	0.67	0.38	0.58	2.05	77.45	Stramenopiles; Chrysophyceae; Chromulinales; JBNA46
U800	0.67	0.38	0.58	2.05	79.5	Rhizaria; Cercozoa; Endomyxa; Vampyrellidae; uncultured
E5	0.67	0.38	0.58	2.05	81.55	Stramenopiles; Chrysophyceae; LG01-09

Only shown are OTUs contributing up to 80% of the cumulative (Cum.) contribution (Contr.).

**Table A2:** **Shared OTUs from alkaline sites determined by SIMPER analysis**.

**OTU ID**	**Ave. abun**	**Ave. sim**	**Sim/SD**	**Contr. %**	**Cum. %**	**Taxonomy**
E81	1	0.61	3.44	6.14	6.14	Stramenopiles; Diatomea; Bacillaryophytina; Fragillariophyceae
E112	1	0.61	3.44	6.14	12.29	Alveolata; Ciliophora; Oligohymenophorea; Peniculia; *Frontonia*
E7	1	0.61	3.44	6.14	18.43	Opisthokonta; Metazoa; Arthropoda; Crustacea; Maxillopoda
E492	0.75	0.33	0.86	3.28	21.71	Alveolata; Ciliophora; Intramacronucleata; Litostomatea; Haptoria; *Lacrymaria*
E723	0.75	0.3	0.87	3.03	24.74	Stramenopiles; Diatomea; Bacillariophytina; Bacillariophyceae; *Sellaphora*
E759	0.75	0.3	0.87	3.03	27.77	Stramenopiles; Diatomea; Bacillariophytina; Bacillariophyceae; *Amphora*
E1947	0.75	0.3	0.87	3.03	30.8	Opisthokonta; Holozoa; Choanomonada; Craspedida
E298	0.75	0.23	0.91	2.36	33.16	Alveolata; Ciliophora; Intramacronucleata; Spirotrichea; Hypotrichia; *Amphisiella-like*
U22	0.75	0.23	0.91	2.36	35.52	Rhizaria; Cercozoa; Thecofilosea
E893	0.75	0.23	0.91	2.36	37.88	Metazoa; Arthropoda; Crustacea; Maxillopoda; Copepoda; Cyclopoida; Cyclopidae
E2036	0.75	0.23	0.91	2.36	40.24	Excavata; Discoba; Kinetoplastea; Eubodonida; *Bodo*
U20	0.5	0.15	0.41	1.47	41.7	Opisthokonta; Metazoa; Arthropoda; Crustacea; Branchiopoda; Artemiidae; *Artemia*
E61	0.5	0.13	0.41	1.27	42.98	Excavata; Discoba; Discicristata; Heterolobosea; Tetramitia; *Naegleria*
E305	0.5	0.13	0.41	1.27	44.25	Alveolata; Ciliophora; Intramacronucleata; Spirotrichea; Hypotrichia; *Oxytrichia*
E391	0.5	0.1	0.41	1.05	45.29	Stramenopiles; Chrysophyceae; Chromulinales; *Spumella*-like
E5	0.5	0.1	0.41	1.05	46.34	Stramenopiles; Chrysophyceae; LG01-09
E3	0.5	0.1	0.41	1.05	47.39	Alveolata; Dinoflagellata; Dinophyceae
E813	0.5	0.1	0.41	1.05	48.43	Alveolata; Ciliophora; Intramacronucleata; Oligohymenophorea; Scuticociliatia
E209	0.5	0.09	0.41	0.88	49.32	Excavata; Discoba; Discicristata; Euglenozoa; Kinetoplastea; Neobodonida
E15	0.5	0.09	0.41	0.88	50.2	Archaeplastida; Chloroplastida; Charophyta; Streptophyta; Tracheophyta

Only shown are OTUs contributing up to 50% of cumulative (Cum.) contribution (Contr.).

**Table A3:** **Indicator “OTU” analysis showing indicator values for representative groups**.

**OTU ID**	**Group**	**IV**_obs_	**Mean**	**S.Dev**	***p***	**Taxonomy**
E19	Acidic	100	51.9	17.84	0.0246^*^	Stramenopiles; Bacillariophyceae; *Pinnularia*
E112	Alkaline	100	64	18.79	0.0246^*^	Alveolata; Ciliophora; *Frontonia*
E928	Acidic	100	44.9	19.72	0.0246^*^	Opisthokonta; Holozoa; Filasterea
U2611	Acidic	100	49	18.68	0.0246^*^	Opisthokonta; Fungi; environmental samples
E7	Alkaline	94	64.2	16.32	0.1102	Opisthokonta; Metazoa; Arthropoda; Crustacea; Maxillopoda
E263	Acidic	66.7	38.5	15.57	0.133	Archaeplastida; Chlorophyta; *Chlamydomonas*
E818	Acidic	66.7	39.8	14.52	0.133	Archaeplastida; Chlorophyta; *Nannochloris*
U105	Acidic	66.7	34.3	18.58	0.133	Archaeplastida; Chlorophyta; *Chloromonas*
E157	Acidic	66.7	34.3	18.78	0.133	Alveolata; Ciliophora; Litostomatea; Haptoria; *Balantidion*
E298	Alkaline	75	52	17.94	0.139	Alveolata; Ciliophora; Spirotrichea; Hypotrichia; *Amphisiella-like*
E893	Alkaline	75	45.3	18.98	0.139	Metazoa; Copepoda; Cyclopoida; Cyclopidae
E2036	Alkaline	75	48.2	19.07	0.139	Excavata; Discoba; Kinetoplastea; Eubodonida; *Bodo*
E723	Alkaline	75	43.6	19.13	0.1454	Stramenopiles; Diatomea; Bacillariophyceae; *Sellaphora*
E759	Alkaline	75	46.7	20.06	0.1454	Stramenopiles; Diatomea; Bacillariophyceae; *Amphora*
E1947	Alkaline	75	44.5	19.07	0.1454	Opisthokonta; Holozoa; Choanomonada; Craspedida
E836	Acidic	66.7	34.6	19.19	0.1458	Opisthokonta; Fungi; Nucletmycea; *Nuclearia*
E1622	Acidic	66.7	39.8	15.08	0.1474	Opisthokonta; RT5iin14
E1833	Acidic	66.7	34.8	19.04	0.1474	Archaeplastida; Chlorophyta; *Chloromonas*

Significant OTUs are indicated with an asterisk.

**Table A4:** **Taxonomy for OTUs listed in Figure 7**.

**OTU ID**	**Taxonomy**
E1035	Opisthokonta; Metazoa; Porifera; Porifera; Calcarea
E1576	Opisthokonta; Metazoa; Arthropoda; unknown
E1821	Excavata; Discoba; Discicristata; Euglenozoa; Kinetoplastea; Neobodonida
E1993	Alveolata; Ciliophora; Intramacronucleata; Oligohymenophorea; Scuticociliatia; *Cohnilembus*
E2131	Alveolata; Ciliophora; Intramacronucleata; Conthreep; Oligohymenophorea; Peritrichia
E2479	Opisthokonta; Metazoa; Arthropoda; Crustacea; Malacostraca
E2855	Alveolata; Ciliophora; Intramacronucleata; Spirotrichea; Hypotrichia; Spirotrachelostyla
E33	Opisthokonta; Metazoa; Cnidaria; Cnidaria; Hydrozoa
E4065	Opisthokonta; Metazoa; Platyhelminthes; Turbellaria; Rhabdocoela
E425	Alveolata; Ciliophora; Intramacronucleata; Conthreep; Phyllopharyngea; Cyrtophoria; AT1-2-like
E523	Opisthokonta; Metazoa; Arthropoda; Crustacea; Cyclopidae
E601	Alveolata; Ciliophora; Postciliodesmatophora; Heterotrichea; unknown
E661	Opisthokonta; Metazoa; Mollusca; Gastropoda; Vetigastropoda
E858	Opisthokonta; Metazoa; Porifera; Porifera; Calcarea
U16709	Apusozoa; Apusomonadidae; *Amastigomonas*
U2762	Alveolata; Ciliophora; Intramacronucleata; Conthreep; Oligohymenophorea; Apostomatia
U302	Opisthokonta; Metazoa; Platyhelminthes; Turbellaria; Seriata; unknown
U3335	Amoebozoa; Discosea; Flabellinia; Stygamoebida; *Vermistella*
U412	Rhizaria; Cercozoa; environmental samples
U73	Opisthokonta; Metazoa; Arthropoda; unknown
**E19**	**Stramenopiles; Diatomea; Bacillariophytina; Bacillariophyceae; *Pinnularia***
E836	Opisthokonta; Fungi; Nucletmycea; *Nuclearia*
**E928**	**Opisthokonta; Holozoa; Filasterea**
**U2611**	**Opisthokonta; Fungi; environmental samples**
E1947	Opisthokonta; Holozoa; Choanomonada; Craspedida
E391	Stramenopiles; Chrysophyceae; Chromulinales; *Spumella*-like
E723	Stramenopiles; Diatomea; Bacillariophytina; Bacillariophyceae; *Sellaphora*
E759	Stramenopiles; Diatomea; Bacillariophytina; Bacillariophyceae; *Amphora*
E813	Alveolata; Ciliophora; Intramacronucleata; Conthreep; Oligohymenophorea; Scuticociliatia
**E112**	**Alveolata; Ciliophora; Oligohymenophorea; *Frontonia***

OTUs most influential in the ordination are color coded: green, Lost City; orange, Rio Tinto, Davis Mine, and Nymph Creek; blue, Mono Lake and Between Tree Claim and Smith; and violet, Mallard Lake. pH extreme indicator OTUs are bolded.
